# Supplementary material for: Description of the Fifth New Species of Russula subsect. Maculatinae from Pakistan Indicates Local Diversity Hotspot of Ectomycorrhizal Fungi in Southwestern Himalayas
Source: Life (Basel). 2021 Jul 6;11(7):662. doi: 10.3390/life11070662 (PMC8303804; doi:10.3390/life11070662)
Supplement: Supplementary file 1 [file life-11-00662-s001.zip › life-1277415-supplementary.pdf]

Supplementary Table S1. Specimens and GenBank accession numbers of DNA sequences used in the phylogenetic analysis. All sequences of R. ayubiana were produced in this study.

Taxon	*Voucher collection (herbarium)*	*Country*	*mtSSU*	*LSU*	*RPB2*	*TEF1α*
R. adulterina	SAV F-4451	Poland	MG386730		MG386750	
R. abbottabadensis	LAH 310099	Pakistan	MG386718		MG386736	
R. abbottabadensis	LAH 310071	Pakistan	MG386719	MN518356	MG386737	MZ364137
R. abbottabadensis	FH 00304558	Pakistan	MG386721	MN518355	MG386738	MZ364138
R. ayubiana	LAH 35438	Pakistan	MZ364121	MZ358816	MZ364131	MZ364139
R. ayubiana	LAH 35439	Pakistan	MZ364122	MZ358817	MZ364132	MZ364140
R. cuprea	GENT (2010 BT168)	Germany	MG386731	MZ358818	MG386751	
R. dryadicola	TURA 151632	Finland	MG386728	MN518357	MG386745	MZ364141
R. dryadicola	TURA 152390	Finland	MG386729	MZ358820	MG386746	MZ364142
R. dryadicola	UPS (at2004140)	Sweden	MZ364125	MN518358	MG386747	MZ364143
R. dryadicola	IB 2002/0432	Italy	MG386722	MZ358819	MG386739	
R. globispora	SAV (HK12021)	Sweden	MZ364123	MN518362	MZ364133	MZ364144
R. globispora	GENT (2007 BT121)	Germany	MG386726	MG944274	MG386743	MZ364145
R. globispora	GENT (2007 BT98)	Germany	MG386727	MZ358822	MG386744	MZ364146
R. globispora	GENT (2010 BT188)	Germany	MZ364124	MZ358821	MZ364134	MZ364147
R. juniperina	SAV F-4998	Italy			MG386749	MZ364148
R. maculata	SAV F-2130	Estonia	MG944263	MG944277	MG944253	
R. maculata	SAV F-933	Slovakia	MG944262	MG944275	MZ364135	
R. mansehraensis	HUP SUR 180	Pakistan	MG944266	MG944280	MG944255	
R. mansehraensis	HUP SUR 803	Pakistan	MG944267		MG944256	
R. mattiroloana	KRA F-2018-1	Poland	MZ364126	MK105723	MZ364136	MZ364149
R. mattiroloana	KRA F-2018-2	Poland	MZ364127	MK105724	MK102759	MZ364150
R. mattiroloana	GK8136	Greece		MK105722	MK102758	
R. mediterraneensis	MCVE 29086 (MG636)	Italy	MZ364128	MZ358824	MK102761	MZ364151
R. mediterraneensis	MCVE 29085 (MG630)	Italy	MZ364129	MZ358823	MK102760	
R. nympharum	M (HM R-12180)	France	MG944270	MG944283	MG944259	
R. nympharum	STU (HJB10019)	Belgium	MZ364130	MN518353	DQ421956	
R. quercus-floribundae	LAH 36219	Pakistan	MN053397	MN513043	MN053389	MZ364152

R. quercus-floribundae	LAH 36220	Pakistan	MN053396	MN513043	MN053390	MZ364153
R. tengii	HMAS 264837	China	MG386733	MN518360	MG386753	MG386707
R. tengii	HMAS 262728	China	MG386734		MG386754	MZ364154
R. tengii	HMAS 244255	China	MG386735		MG386755	MZ364155
R. tengii	HMAS 251829	China	MG386732	MN518359	MG386752	MZ364156
